# Compressive Failure Mechanisms of NCF Laminates with Double-Hole Defects

**DOI:** 10.3390/ma19030495

**Published:** 2026-01-26

**Authors:** Songming Cai, Shi Yan, Lili Jiang, Zixiang Meng, Yongxin Niu

**Affiliations:** 1 Architectural Engineering Institute, Harbin University of Science and Technology, Harbin 150080, China; 2 School of Civil Engineering and Architecture, Xiamen City University, Xiamen 361008, China

**Keywords:** composite laminates, open-hole compression, finite element modeling, damage evolution

## Abstract

**Highlights:**

**What are the main findings?**

- OHC strength shows a spacing threshold: changes beyond s/D ≥ 3 are small.
- At s/D = 2, transverse alignment is the weakest; direction sensitivity diminishes at larger spacing.
- Apparent elastic stiffness stays nearly constant across all nine configurations (≤~1% variation).

**What are the implication of the main findings?**

- Design-wise, increasing spacing from s/D = 2 → 3 is the key gain; further spacing gives diminishing returns.
- NCF stitching/drilling defects can promote ligament damage coalescence at small spacing, so narrow ligaments should be avoided.
- The FE framework (3D Hashin + shear-coupled matrix compression + cohesive delamination) reproduces response and failure mechanisms.

**Abstract:**

Open-hole compression (OHC) tests were carried out on non-crimp fabric (NCF) laminates with varied open-hole orientation (angle to the loading direction) and inter-hole spacing. Failure modes were documented by scanning electron microscopy (SEM), and the compressive strength was quantified. Finite element simulations in Abaqus were developed to replicate the tests, establishing a progressive-damage model for open-hole laminates under compression. Intralaminar failure was described using the three-dimensional Hashin failure criterion and a modified matrix compression criterion incorporating shear coupling effects, while interlaminar delamination was modeled with cohesive elements, enabling the simulation of damage initiation, growth, delamination, and final collapse. The results show that hole orientation and spacing have a pronounced effect on open-hole compression (OHC) strength. A spacing threshold is observed, beyond which further increases in spacing provide little additional benefit. In contrast, the apparent elastic stiffness is essentially insensitive to hole spacing and orientation. The combined intralaminar and interlaminar model successfully reproduces the characteristic mechanical response—linear elasticity followed by catastrophic failure—in good agreement with the experiments.

## 1. Introduction

Non-crimp fabric (NCF) consists of parallel, non-interlaced fiber tows fixed by stitching or bonding. The fibers remain straight, which reduces fiber crimp and the associated loss of mechanical properties. NCF laminates achieve high strength and stiffness through multilayer reinforcement (e.g., 0°, ±45°, 90°) and typically offer a high fiber volume fraction. Compared with woven fabrics, NCFs provide superior mechanical performance, better formability, and higher processing efficiency. As a result, NCFs are widely used in aerospace, automotive, wind energy, and other industrial structures [1,2,3,4]. However, the presence of through-thickness stitching yarns, while stabilizing fiber tows, introduces inherent heterogeneity. The stitching points often create resin-rich pockets (meso-scale defects), which can act as stress concentrators. Furthermore, during mechanical machining processes such as drilling, the stitching yarns are severed at the hole boundary. The literature [5,6] suggests that this severance can induce specific damage modes, such as stitch pull-out and localized delamination initiated from the loosened fiber bundles, potentially negating the through-thickness reinforcement benefit usually associated with stitching. However, holes introduce stress concentrations and affect the overall mechanical properties of the material. Especially for NCF composites, the stress concentration around the opening is more significant, which may lead to local damage or failure [7,8]. Hole geometry—size, spacing, shape, and orientation—strongly influences ultimate load capacity, compressive strength, fatigue life, and other laminate properties [9,10]. Therefore, understanding how holes affect mechanical performance guides structural optimization and performance enhancement in composite structures. This study investigates the influence of hole spacing and two-hole orientation on the OHC response of NCF laminates with a double-hole defect, using combined experiments and finite element simulations to simulate non-crimp fabric (NCF) open-hole compression tests.

Researchers systematically analyzed the mechanics of composite open-hole laminates using experiments and simulations. Su et al. [11] proposed a progressive-damage model for open-hole compression (OHC) and applied it to study size effects. The framework employed continuous shell elements to capture in-plane and out-of-plane deformation, cohesive elements to model delamination, and a smeared-crack approach to represent progressive failure. Their results showed that the model predicted laminate strength and failure modes with good accuracy. Saeed et al. [12] performed combined-loading compression (CLC) tests on pristine laminates and conducted post-impact compression-after-impact (CAI) and OHC tests on damaged laminates. Fracture morphology was examined by scanning electron microscopy (SEM). The results showed that the weakening effect of impact damage on compressive strength is stronger than that of open-hole defects. Final failure in both specimen types was governed by fiber micro-buckling within kink bands, interlaminar shear, and matrix/fiber microcracking; longitudinal splitting promoted early crack growth and accelerated failure progression. Takamoto et al. [13] investigated the effects of ply thickness and stacking sequence on OHC behavior in thin CFRP laminates. They experimentally quantified the influence of these parameters on OHC strength and simulated damage progression using finite element analysis with the Hashin 2D failure criterion for intralaminar damage and a cohesive-zone model for delamination. Shimizu et al. [14] investigated how stress concentration influences compressive failure in perforated carbon-fiber-reinforced polymer (CFRP) laminates using combined experiments and simulations. The relationship between failure behavior and stress concentration was discussed by changing the aspect ratio of the elliptical hole. Numerical predictions agreed well with experiments across aspect ratios for the stresses at initial damage and at final failure. The simulated damage evolution in the 0° plies was also highly consistent with the observations. Leveraging the simulations, they further examined internal damage and delamination mechanisms that are difficult to observe experimentally. Soutis [15] conducted impact tests and (CAI) experiments on carbon-fiber/epoxy laminates. A cohesive-zone model was employed to estimate the post-impact compressive strength. The model predicted residual strength across different fiber systems and stacking sequences with errors typically below 10%. Rodríguez-Sereno et al. [16] investigated how holes influence the dynamic compressive response of CFRP laminates. They showed that a maximum-stress criterion identified the onset of transverse shear failure in the braided composite more accurately. Building on this, they analyzed open-hole specimens of the same material and found that, when fibers were aligned with the loading direction, the hole produced a more pronounced reduction in strength. Özaslan [17] investigated stress fields and the strength of carbon-fiber/epoxy (CFRP) laminates with oriented holes using combined experimental tests and numerical analysis. They examined hole–hole interaction and analyzed how hole position and loading direction affected the laminate’s stress distribution. In addition, they proposed an extended point-stress criterion (EPSC) to predict the failure loads of specimens with oriented holes and validated the approach experimentally. Solis et al. [18,19] analyzed interlaminar stresses in open-hole CFRP laminates under compressive loading using a numerical framework that combined a series–parallel hybrid theory to estimate laminate stiffness from constituent properties with a continuum damage mechanics (CDM) model to capture damage initiation driven by fiber micro-buckling. Predictions were validated against published experiments and reproduced the global response. The simulations indicated that damage initiated at the laminate mid-plane—particularly in the thickest ply aligned with the loading direction—and then propagated into neighboring plies of the same orientation. Two stacking sequences were compared. Around the hole, interlaminar stresses were symmetric about the loading and transverse axes, and the extrema of individual stress components occurred at different circumferential angles for each laminate. Suemasu et al. [20] investigated compression failure mechanisms in open-hole, quasi-isotropic composite laminates using experiments and numerical simulations and related the observed failure behavior to material properties. Experiments indicated that fiber micro-buckling in the 0° plies initiated damage. In laminates with high interlaminar toughness, damage accumulated gradually and culminated in interlaminar delamination prior to final fracture, whereas laminates with low interlaminar toughness failed more abruptly. Numerical analyses showed that micro-buckling-induced stress redistribution and damage accumulation evolved as the number of damaged elements increased, ultimately producing a stable, progressive-damage path. Komur et al. [21] investigated the buckling behavior of laminated composite plates with circular and elliptical holes. Using the finite element method (FEM), they conducted a parametric study with varying hole shapes and positions to evaluate their effects on the critical buckling load of square plates. Zhou et al. [22] conducted compression tests on open-hole composite laminates with different stacking sequences and documented specimen strength and failure modes. They developed an ABAQUS-based progressive-damage model that combined multiple failure criteria to simulate damage initiation, propagation, and ultimate failure. The model predicted laminate strength and failure modes and was used to assess how the stacking sequence influenced the ultimate failure pattern. The modeling framework was grounded in Micromechanics of Failure (MMF) theory. Wang et al. [23] investigated progressive failure in composite structures. They built a unit-cell model representing a face-centered array of carbon fibers, examined microscale stress amplification in the fibers and matrix, and proposed a noninteractive failure criterion to identify failure initiation. Coupling ABAQUS user-defined material subroutines (UMAT/VUMAT) with Fortran scripts, they simulated failure evolution and property degradation of the composites. Comparisons with experiments verified the approach for predicting OHC strength.

Despite extensive work on damage analysis of composite laminates—including numerous models, experimental criteria, and small representative volume elements (RVEs)—few studies have examined how hole orientation (opening angle) and hole spacing influence compressive performance. To address this gap, this study considered nine open-hole configurations to evaluate their effects on the compressive mechanical properties and failure modes of the specimens. Specimens were CNC-machined and then subjected to quasi-static axial compression; microstructural damage was examined by SEM. ABAQUS was used to simulate the non-crimp fabric (NCF) OHC tests. A user-written VUMAT implemented the elastic response, damage evolution, and failure checks for orthotropic composites, following the 3D Hashin and a modified matrix compression criterion incorporating shear coupling effects. An interlaminar damage criterion was incorporated to capture delamination propagation and the associated failure modes. Simulation results were compared with experiments to validate the model.

## 2. Theoretical Background

### Mechanics of Multi-Hole Interference Effects

The mechanical response of laminates containing multiple discontinuities is governed by the superposition of stress fields. For an orthotropic laminate containing two circular holes of diameter D spaced at a center-to-center distance s, the effective Stress Concentration Factor (SCF) is a function of the spacing ratio s/D and the alignment angle θ. According to the complex potential theory for anisotropic plates [24], the stress perturbation around a single hole decays at a rate proportional to (D/r)^n^. When the spacing s is sufficiently small, the perturbation zones of adjacent holes overlap, creating a “compound interference zone.”

Under compressive loading, this interference manifests through two primary mechanisms:

(1) Stress Amplification: The peak tangential stress at the hole edge is elevated due to the proximity of the second stress singularity. This effect is particularly pronounced in transverse configurations (θ = 90°), where the interacting stress fields create a high-stress corridor within the ligament.
(2) Ligament Instability: The material ligament between holes undergoes accelerated micro-buckling or shear collapse. Due to the constrained volume, stress redistribution is inhibited, leading to premature instability compared to an isolated hole [25].

Theoretically, the transition from “interactive failure” to “independent failure” corresponds to the decay of the interaction factor Y. In isotropic elasticity, the interaction is often considered negligible when the spacing ratio reaches s/D ≥ 3 [26]. However, for NCF composites, the inherent heterogeneity introduced by stitching loops defines a larger characteristic damage process zone (dp). We hypothesize that significant strength degradation occurs when the ligament width falls below a critical threshold where the damage zones of adjacent holes coalesce (i.e., s < D + 2 dp). This theoretical framework underpins the experimental investigation into the specific threshold behavior of NCF laminates presented in this study.

## 3. Experiments

### 3.1. Materials and Equipment

**Figure 1:**
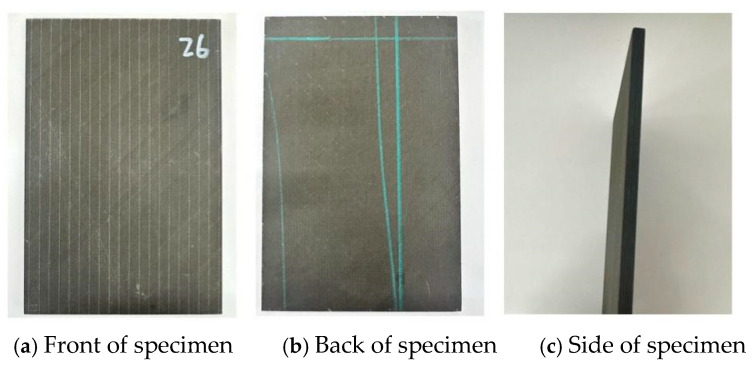
NCF composite specimens.

NCF composite laminates were used in this study. The carbon fiber used was CCF300 3k (T300 class) supplied by Weihai TuoZhan Fiber Co., Ltd., Weihai, China. (equivalent to Toray T300 3k), and the matrix material was TED-86 epoxy resin. The laminate layup is [+45°/0°/90°/−45°] _s_, the specimen size is 150 mm × 100 mm × 5 mm, and the measurement error was controlled to within 0.2 mm. The upper and lower surfaces and sides of the specimen are shown in Figure 1, and related parameters are shown in Table 1.

### 3.2. Experimental Method

**Figure 2:**
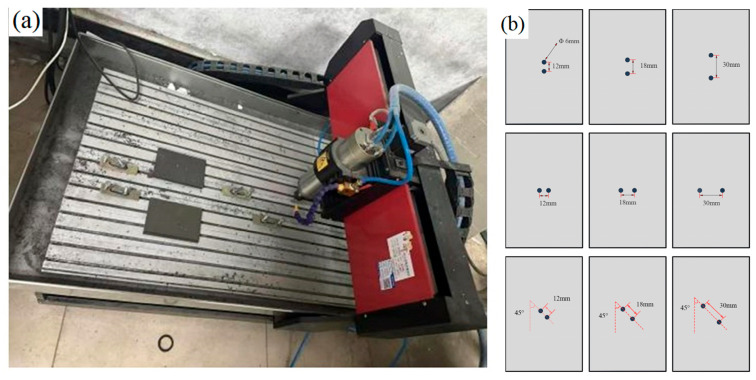
(**a**) CNC engraving machine. (**b**) Specimen opening diagram.

The openings were machined using a YD6090-G computer numerical control (CNC) engraving machine (Jinan Yidiao CNC Equipment Co., Ltd., Jinan, China), and subsequent OHC tests were conducted on a universal testing machine (MTS Systems Corporation, Eden Prairie, MN, USA) with a maximum load capacity of 2000 kN. From compression tests conducted at a compression speed of 0.5 mm/min, the macroscopic axial compressive stress–strain curve was obtained. Compressive damage was characterized by scanning electron microscopy (SEM) (Hitachi SU5000, Hitachi High-Technologies Corporation, Tokyo, Japan), and the tests were subsequently simulated in Abaqus/Explicit 2023 (Dassault Systèmes, Vélizy-Villacoublay, France). The diameter of the drill used in the CNC engraving machine is 6 mm. The spindle speed was set to 8000 rpm. The CNC engraving machine is shown in Figure 2a. Three replicates were prepared for each configuration. The open-hole design combined three levels of hole spacing with three orientation angles (a 3 × 3 factorial), yielding nine distinct opening configurations. The three two-hole orientation angles were θ = 0° (longitudinal), 45°, and 90° (transverse), measured counterclockwise from the specimen’s longitudinal axis. A schematic of the hole layout is shown in Figure 2b.

The typical specimen numbers are shown in Table 2. Two control groups were included for comparison.

### 3.3. Compression Test

It is important to quantify the influence of the side-clamped boundary conditions (ASTM D7137 [27]) utilized in this study compared to the standard side-supported OHC method (ASTM D6484 [28]). The rigid side clamping imposes a transverse displacement constraint (ε_yy_ = 0) at the specimen edges. According to laminate theory, this induces a secondary transverse compressive stress σ_yy_ = ν_xy_$\frac{E_{yy}}{E_{xx}}$σ_xx_ due to the Poisson effect. For the quasi-isotropic NCF laminate used here, this confinement creates a partial bi-axial compression state in the far field. While this boundary condition tends to slightly elevate the measured ultimate compressive strength by suppressing premature edge splitting and global buckling, it forces damage initiation and propagation to localize exclusively within the hole interaction zone. Therefore, while a direct comparison of absolute strength values with the standard OHC literature requires consideration of this confinement effect, the relative trends regarding hole spacing thresholds and interaction mechanisms remain valid and intrinsic to the material response.

**Figure 3:**
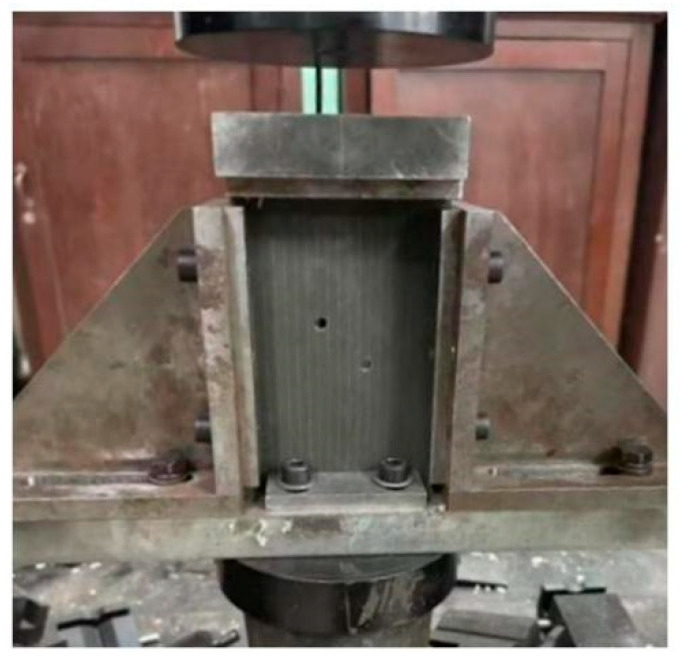
Compression test setup.

A purpose-built compression fixture was designed to accommodate the specimen geometry. To suppress global buckling of the large thin plate (150 mm × 100 mm) and stabilize the observation of near-hole damage evolution, axial compression was performed using a side-clamped anti-buckling fixture based on the ASTM D7137 [27] standard. It is acknowledged that this setup imposes stronger lateral constraints compared to the standard open-hole compression (OHC) test (ASTM D6484 [28]), which typically uses a side-supported or unsupported gauge section. However, given the thin-plate geometry, the anti-buckling support was deemed necessary to ensure that failure was driven by hole edge stress concentrations rather than structural instability. While this boundary condition may slightly elevate the apparent strength due to Poisson effect constraints, it allows for a consistent comparative analysis of hole spacing effects under controlled conditions. A schematic of the compression setup is shown in Figure 3.

Tests were conducted on an MTS 2500 universal testing machine (MTS Systems Corporation, Eden Prairie, MN, USA). The system had a rated capacity of 200 kN, the load accuracy was ±1%, and the displacement resolution was 0.01 mm. The testing system automatically recorded the load–displacement response, from which the engineering stress–strain curve was obtained. Ultimate compressive strength was calculated using Equation (1), and Young’s modulus was taken as the slope of the initial linear portion of the engineering stress–strain curve. During testing, the specimen was aligned vertically, with its longitudinal axis coincident with the loading axis. Displacement control was used at a crosshead rate of 0.5 mm/min and continued until specimen crushing.

(1) $$\sigma =\frac{F}{A}$$

In Equation (1), *σ* denotes the ultimate compressive stress, *F* is the maximum load sustained by the specimen, and *A* is the loaded cross-sectional (contact) area of the specimen.

## 4. Finite Element Analysis

### 4.1. Finite Element Model

**Figure 4:**
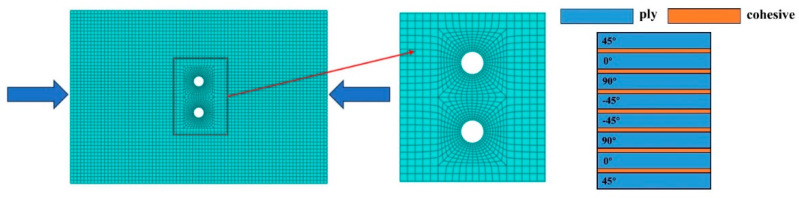
Meshing and layering diagram.

The NCF laminate was modeled using 3D continuum (solid) elements. A locally refined mesh was used around the opening to capture the stress concentration. The laminate plies were modeled with C3D8R solids and zero-thickness COH3D8 cohesive elements at every ply interface. A locally refined mesh was used around each hole and across the inter-hole ligament, with a coarser mesh elsewhere; at least one solid layer per ply was used through the thickness (cohesive inserted at all interfaces). Mesh convergence was checked by h-refinement in the refined patch: the peak load changed by ≤2%, and the distributions of damage variables (e.g., SDEG and intralaminar damage) were invariant within ≤5%. These thresholds were taken as sufficient convergence. An equivalent boundary treatment was used to mirror the fixture: nodes within the 12.5 mm clamped margins were kinematically coupled to two rigid reference points/analytical rigid surfaces on the specimen sides, with hard contact and clamping pressure enforcing out-of-plane suppression while permitting in-plane sliding. No additional in-plane constraints were applied inside the window to avoid over-constraint. The bottom reference point was fixed, and a displacement matching the tests (0.5 mm/min) was prescribed at the top reference point. The intralaminar elastic constants and strengths were measured on dedicated coupons following the relevant standards (tension/compression/shear), and the interlaminar fracture energies were identified from DCB/ENF/mixed-mode tests. The mean values were used in the simulations. The material density was ρ = 1.8 × 10^−9^ t/mm^3^. The orthotropic elastic moduli were E_1_ = 133.4 GPa and E_2_ = E_3_ = 7.2 GPa; Poisson’s ratios were ν_12_ = ν_13_ = 0.26 and ν_23_ = 0.40; and the shear moduli were G_12_ = G_13_ = 2.8 GPa and G_23_ = 1.3 GPa. Damage initiation and evolution parameters are listed in Table 3. The finite element mesh and layup are shown in Figure 4.

This study investigates carbon-fiber/epoxy NCF laminates and establishes a finite element model in Abaqus/Explicit using 3D solid (C3D8R) elements. Intralaminar damage was implemented in a user-defined material subroutine (VUMAT) that combined the 3D Hashin criterion with a modified matrix compression formulation incorporating shear–compression coupling effects. Stiffness degradation was driven by damage variables. In addition, interlaminar failure (delamination) was considered. Fiber tension/compression failure was evaluated by the 3D Hashin model, whereas matrix cracking/crushing under multiaxial stress was assessed by a modified quadratic criterion incorporating shear–compression coupling effects, accounting for both fiber-parallel and transverse responses. Damage evolution was tracked via history variables, and the constitutive stiffness matrix was updated accordingly. At each increment, the subroutine read the material constants (elastic moduli, Poisson’s ratios, strengths), updated strains from the kinematics, computed stresses with the current degraded stiffness, evaluated the failure indices, updated the damage variables, further degraded the stiffness as required, and returned the updated stress state.

### 4.2. Rationale for Using Abaqus/Explicit and VUMAT

Under compressive loading, the laminate exhibits pronounced softening, localization, and contact (with the side anti-buckling fixture and at the hole edges), for which implicit formulations tend to become unstable or fail to converge during damage evolution and in the post-peak regime. By contrast, an explicit central-difference scheme is non-iterative and remains robust under severe nonlinearity and contact; coupled with a user-defined material subroutine (VUMAT), it enables an increment-by-increment, integration-point-level sequence of stress update → failure checks → stiffness degradation, facilitating the implementation of progressive-damage models such as 3D Hashin and the modified matrix criterion with shear coupling, and their coupling with cohesive elements.

To obtain a quasi-static response within the explicit framework, we applied a low-rate, smooth displacement control consistent with the experiments (equivalent to 0.5 mm/min). To ensure the validity of the quasi-static assumption, the energy balance was rigorously monitored. The ratio of kinetic energy (ALLKE) to internal energy (ALLIE) was maintained below 5% during the major damage evolution phase, confirming that dynamic effects were negligible. Furthermore, the artificial strain energy (ALLAE) utilized for hourglass control was restricted to less than 2% of the total energy, ensuring that numerical artifacts did not distort the physical response. Finally, doubling the total loading time changed the peak load and failure morphology by less than 2%, confirming the rate insensitivity of the model. These checks indicate negligible inertial effects, limited numerical dissipation, and rate insensitivity, confirming that the explicit-solver simulations are quasi-static.

### 4.3. Intralaminar Damage Criterion

The detailed damage criterion can be found in File S1: Damage Criterion. The 3D Hashin failure criterion was implemented via a VUMAT subroutine to predict intralaminar damage initiation. While standard formulations were used for fiber failure and matrix tension, the matrix compression mode was modified to account for the shear–compression coupling effect observed in NCF laminates. The failure indices (F) for the four characteristic modes are defined as follows:

Fiber Tension ($\varepsilon_{11}\geq 0$):

(2) $${F_{ft}=\;\left(\frac{\sigma_{11}}{{Χ}_{T}}\right)}^{2}+{\left(\frac{\sigma_{12}}{S_{12}}\right)}^{2}+{\left(\frac{\sigma_{13}}{S_{13}}\right)}^{2}\geq 1$$

Fiber Compression ($\varepsilon_{11}<0$):

(3) $${F_{fc}=\;\left(\frac{\sigma_{11}}{{Χ}_{C}}\right)}^{2}\geq 1$$

Matrix Tension ($\sigma_{22}+\sigma_{33}\geq 0$):

(4) $${F_{mt}=\;\left(\frac{\sigma_{22}+\sigma_{33}}{Y_{T}}\right)}^{2}+\frac{{\sigma_{23}}^{2}+{\sigma_{12}}^{2}+{\sigma_{13}}^{2}-\sigma_{22}\sigma_{33}}{{S_{23}}^{2}}\geq 1$$

Matrix Compression ($\sigma_{22}+\sigma_{33}<0$):

(5) $${F_{mc}=\;\left(\frac{\sigma_{22}+\sigma_{33}}{2S_{12}}\right)}^{2}+\frac{{\sigma_{23}}^{2}+\left|\sigma_{12}\sigma_{13}\right|}{{S_{23}}^{2}}+\left(\frac{{\sigma_{12}}^{2}+{\sigma_{13}}^{2}}{{S_{12}}^{2}}\right)+\alpha \left(\frac{\sigma_{22}+\sigma_{33}}{S_{12}}\right)\geq 1$$

Here, *σ_ij_* are the components of the effective stress tensor, and *X_T_*, *X_C_*, *Y_T_*, and *S_ij_* denote the respective strength properties. The coefficient α in Equation (5) represents the shear–compression coupling, capturing the internal friction effect where transverse compression suppresses shear band formation. This modification is critical for accurately modeling the failure of NCF laminates, where resin-rich pockets are sensitive to multiaxial stress states.

### 4.4. Interlaminar Damage Model

The interlaminar behavior was modeled using zero-thickness cohesive elements (COH3D8) governed by a bilinear traction–separation law. Damage initiation is predicted using the quadratic nominal stress criterion:

(6) $${\left(\frac{\langle t_{n}\rangle }{N}\right)}^{2}+{\left(\frac{t_{s}}{S_{shear}}\right)}^{2}+{\left(\frac{t_{t}}{S_{shear}}\right)}^{2}\;=\;1$$

where t_n_, t_s_, and t_t_ are the traction components in the normal and two shear directions, respectively. N and S_shear_ represent the interfacial normal and shear strengths. The Macaulay brackets ⟨ ⟩ ensure that purely compressive normal stress does not induce damage initiation.

Once damage initiates, stiffness degradation is governed by an energy-based evolution law. To account for the dependence of fracture toughness on mode mixity, the Benzeggagh–Kenane (B-K) fracture criterion was employed:

(7) $$G_{equiv}^{c}\;=\;G_{IC}+\left(G_{\mathrm{II}C}-G_{IC}\right){\left(\frac{G_{shear}}{G_{T}}\right)}^{\eta }$$

where G_IC_ and G_IIC_ are the critical fracture energies for Mode I and Mode II, G_shear_ = G_II_ + G_III_, G_T_ = G_I_ + G_shear_, and η is the B-K power law parameter determined experimentally.

## 5. Results and Discussion

### 5.1. Damage Propagation and Failure Mechanism

**Figure 5:**
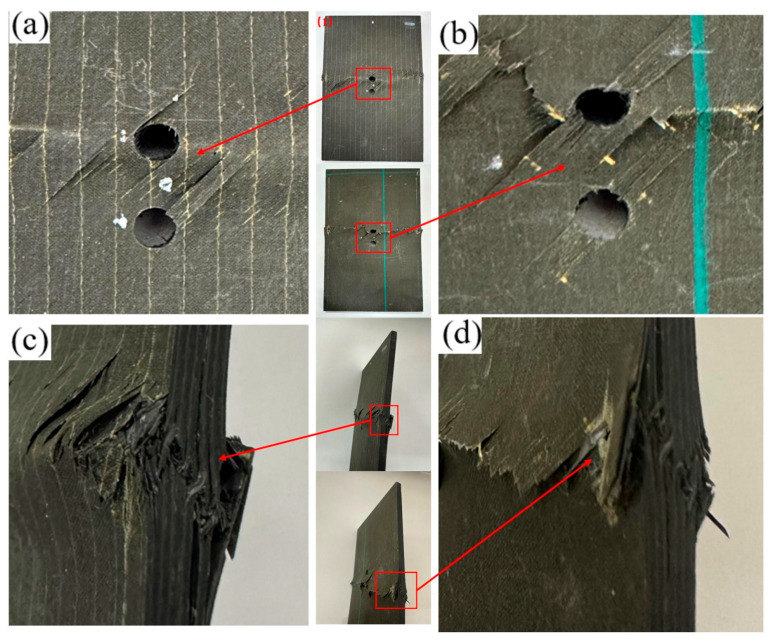
Failure diagram of specimen No. 1 with a 12 mm longitudinal opening. (**a**) Front view of the specimen; (**b**) Back view of the specimen; (**c**) Side view of the specimen edge; (**d**) Enlarged view of the edge damage.

The specimen with an 18 mm opening lies in a transition regime: its failure morphology is intermediate between the two bounding cases and does not introduce a new mechanism. Accordingly, the 18 mm case is not shown. As visible in Figure 5a,b, during loading, the fibers at the hole edge underwent local bending/kinking and fracture; some fibers experienced bearing-induced crushing/indentation followed by partial pull-out. Localized material crushing is observed around the hole; matrix crushing and spallation are evident at the hole edge. These features indicate that stress concentration at the hole boundary amplifies the local damage. A pronounced oblique shear crack is observed in the ligament between adjacent holes. The crack initiates at the hole edge and propagates toward the laminate mid-width, following the fiber direction and preferentially along ply interfaces, manifesting as interlaminar delamination. Fibrillar (filamentous) fracture features are observed in portions of both fibers and the matrix, accompanied by matrix cracking and fiber pull-out. The fracture path is predominantly straight or obliquely oriented. As shown in Figure 5c, both fibers and the matrix exhibit pronounced buckling with material pile-up. Stepped, through-thickness delamination develops, accompanied by local fiber kinking and breakage. These observations indicate that compressive loading drives a severe coupled failure mode—delamination coexisting with fiber failure at the edge. As shown in Figure 5d, interlaminar delamination is the dominant failure feature, accompanied by matrix cracking and fiber fracture. Along the free edge of the laminate, ply separation with upturned laminate is evident, and the delamination front propagates outward from the edge. Table 4 summarizes the failure modes at the respective locations.

**Figure 6:**
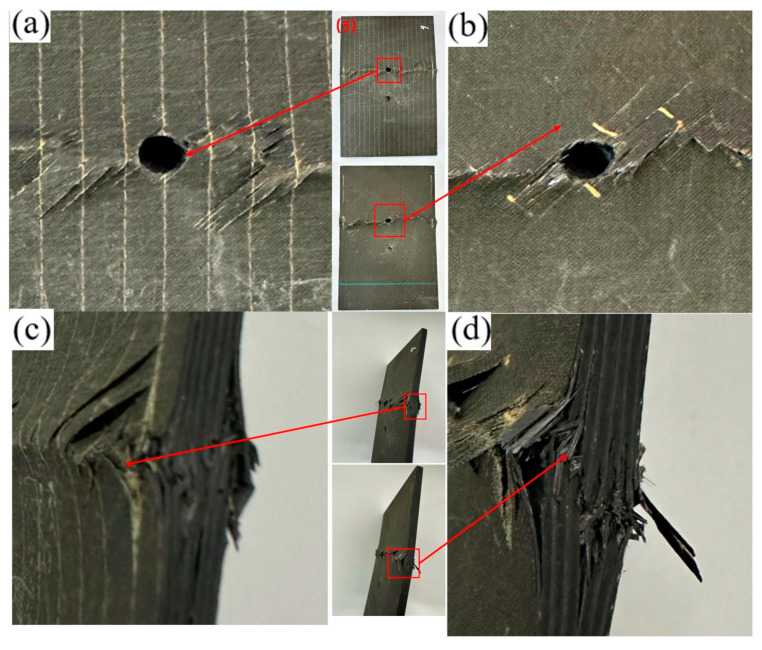
Failure diagram of specimen No. 5 with a 30 mm longitudinal opening. (**a**) Front view of the specimen; (**b**) Back view of the specimen; (**c**) Side view of the specimen edge; (**d**) Enlarged view of the edge damage.

As shown in Figure 6b, with increasing longitudinal hole spacing, the failure mode of the specimen is triggered by the stress concentration around the hole and tends to be initiated at the interlaminar interface or the edge weakness of the plate. The crack peels off layer by layer along the ply interface to form a stepped layer and penetrates the thickness. The bearing failure is limited to the cross-section of the single hole.

**Figure 7:**
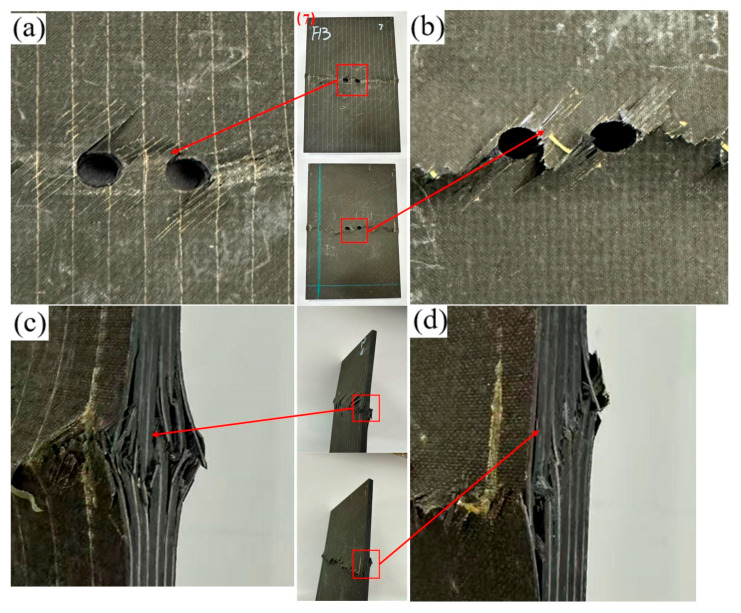
Failure diagram of specimen No. 7 with a 12 mm transverse opening. (**a**) Front view of the specimen; (**b**) Back view of the specimen; (**c**) Side view of the specimen edge; (**d**) Enlarged view of the edge damage.

**Figure 8:**
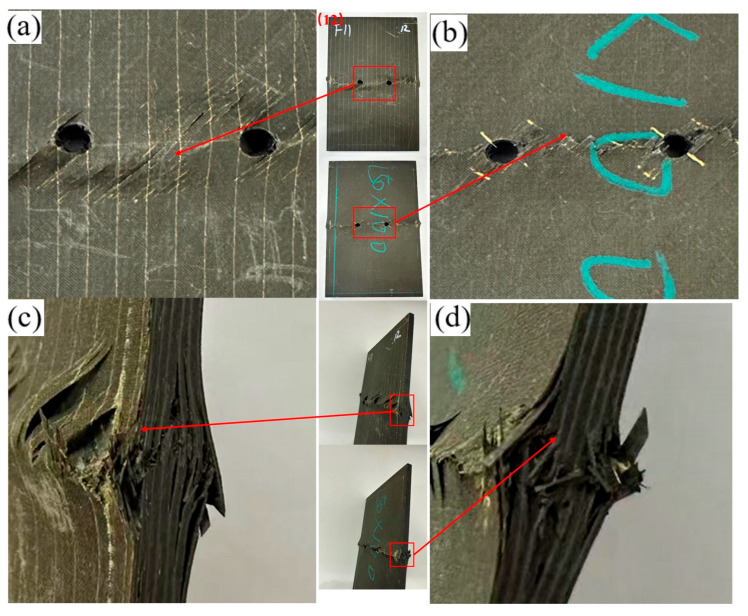
Failure diagram of specimen No. 12 with a 30 mm transverse opening. (**a**) Front view of the specimen; (**b**) Back view of the specimen; (**c**) Side view of the specimen edge; (**d**) Enlarged view of the edge damage.

For the transverse opening configuration (Figure 7 and Figure 8), the overall failure mechanisms are similar to those observed in the longitudinal case (Figure 5 and Figure 6), being dominated by matrix-controlled crushing and shear at the hole edge, accompanied by fiber–matrix interfacial debonding and interlaminar delamination. The hole exhibits slight ovalization, and stepped delamination develops along the side surface. Increasing the hole spacing does not alter the global failure mechanism; however, consistent with the longitudinal configuration, the spatial distribution of damage changes markedly, with strong hole–hole interaction and ligament-spanning damage at small spacings and localized, near-independent damage at larger spacings.

**Figure 9:**
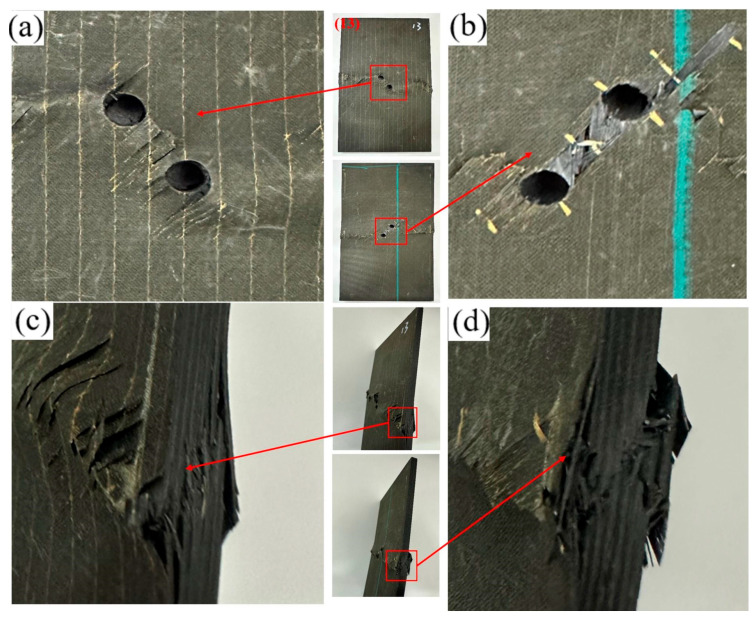
The failure diagram of specimen No. 13 with a hole spacing of 12 mm in the 45° direction. (**a**) Front view of the specimen; (**b**) Back view of the specimen; (**c**) Side view of the specimen edge; (**d**) Enlarged view of the edge damage.

**Figure 10:**
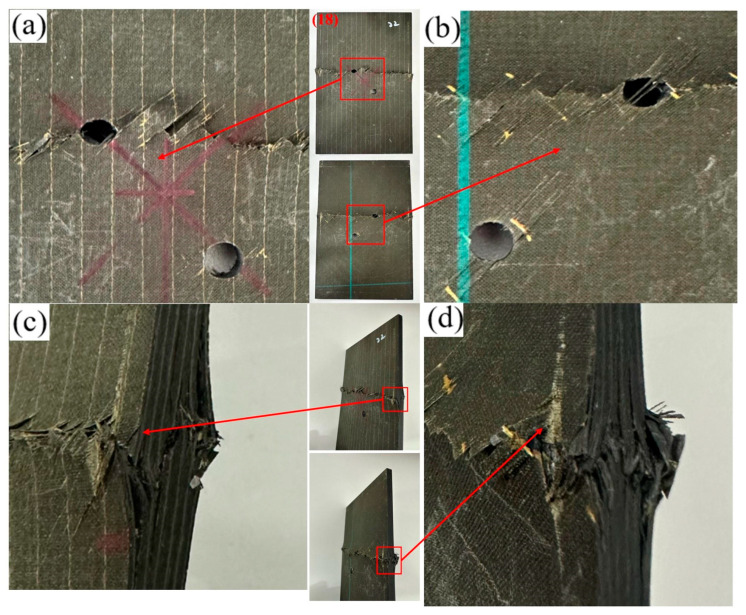
The failure diagram of specimen No. 18 with a hole spacing of 30 mm in the 45° direction. (**a**) Front view of the specimen; (**b**) Back view of the specimen; (**c**) Side view of the specimen edge; (**d**) Enlarged view of the edge damage.

For the 45° opening configuration (Figure 9 and Figure 10), the dominant failure features are generally consistent with those of the longitudinal and transverse configurations (Figure 5, Figure 6, Figure 7 and Figure 8), characterized by matrix-dominated crushing and shear, fiber–matrix interfacial debonding, and extensive interlaminar delamination under compressive loading. In addition, pronounced buckling and material accumulation extend transversely toward the holes, and cracks in the 45° fiber layers preferentially propagate along the fiber direction. As the hole spacing increases to 30 mm, hole–hole interactions are markedly reduced, with the inter-hole ligament remaining largely intact and the cross-hole shear band no longer forming a continuous path, following the same decoupling trend observed in the other two orientations.

**Figure 11:**
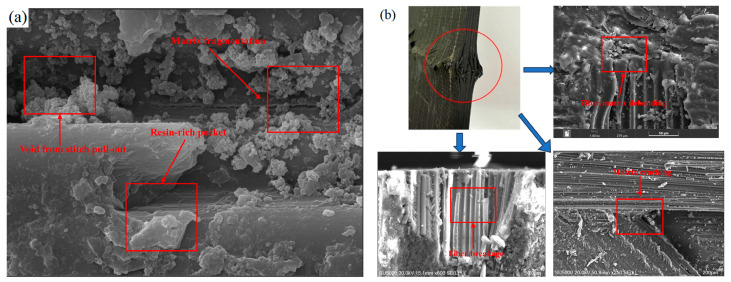
(**a**) SEM micrograph; (**b**) NCF-specific meso-structural defects on the interlaminar fracture surface.

As shown in the SEM micrographs in Figure 11a, damage is dominated by matrix yielding/crushing and fiber micro-buckling (kink bands), accompanied by interlaminar delamination and growth of interfacial debonding—a typical shear–compression coupled failure morphology under OH loading. Fractographic examination reveals pronounced matrix tearing and fragmentation and extensive carbon-fiber tow fracture; overall, the failure is shear-dominated. This arises because the specimen is a two-dimensional laminate, whose overall structural integrity and shear resistance are inferior to those of a three-dimensional braided architecture. During post-impact axial compression, fiber tows misaligned with the loading direction are subjected to higher in-plane shear, making them more susceptible to shear-dominated failure. Additionally, interlaminar bonding relies primarily on matrix adhesion developed during hot-press consolidation, rather than through structural connections. Because both the fiber and matrix exhibit low shear strength, pronounced shear deformation develops during compression. Consequently, a failure mode characterized by fiber-tow fracture and matrix failure is frequently observed.

To specifically address the micromechanical role of the NCF stitching yarns under OHC conditions, a detailed examination of interlaminar fracture regions was conducted, as shown in Figure 11b. Unlike typical prepreg laminates, distinct meso-scale features related to the stitching architecture are evident. Large voids, identified as stitch pull-out holes, indicate that the stitching yarns were physically severed during the hole-making process at the critical hole boundary. Surrounding these voids are resin-rich pockets—artifacts of the stitching process—which exhibit extensive matrix fragmentation. These observations suggest that, due to drilling damage, the stitching points at the hole edge acted as damage initiation sites caused by stress concentration within the brittle resin, rather than providing the through-thickness reinforcement or crack arrest mechanisms typically associated with NCF materials.

#### Influence of Meso-Scale Stitching Defects on Hole Interaction

A unique feature of the observed failure morphology is the interaction between the drilled hole edge and the NCF stitching yarns. As detailed in Figure 11b, the stitching points act as micro-crack initiation sites. In the double-hole configurations with small spacing (12 mm), the overlapping stress fields likely connect these stitching-induced defects across the ligament. Unlike woven fabrics, where crimp dominates, in NCF, the resin-rich zones at stitch locations constitute weak links that facilitate the propagation of transverse shear cracks.

Specifically, when the inter-hole ligament width is comparable to the stitching pitch (approx. 5 mm), the probability of “defect coalescence” increases. This explains the sharp drop in strength for the 12 mm spacing observed in Section 5.2: the effective structural ligament is further compromised by the pre-existing meso-scale voids from stitch severance. This mechanism is distinct from standard laminates and underscores the necessity of avoiding hole placements that create ligaments narrower than 3–4 times the stitch pitch.

Across all three opening-angle configurations, the specimens exhibited matrix-dominated crush–shear failure under axial (longitudinal) compression, accompanied by fiber–matrix interfacial debonding and interlaminar delamination. Analysis indicates that the stress-field interaction between the two holes is strongest along the 45° direction. At the hole’s trailing edge, the shear band and delamination coalesce and propagate through the inter-hole ligament, forming the primary shear band bridging the holes. In the thickness direction, multi-layer delamination and wedge-type tearing occur, resulting in a sudden drop in load after the peak. The two-hole damage field of the longitudinally perforated specimen is approximately independent of each hole, and the inter-hole ligament remains intact, showing better progressiveness as a whole. Among the three configurations, the specimen with transversely aligned holes exhibits an intermediate response: a partial damage bridge forms between the holes but does not develop into a continuous connection. It can be seen that the dominant mechanism of the three groups of specimens is consistent, and the difference is mainly due to the interaction between the two holes. Thus, the failure mode evolves from coupling connectivity to decoupling localization.

### 5.2. Summary of Experimental Results

The compression test results are summarized in Table 5 as the average of the three replicate groups (*n* = 3). It should be noted that the sample size for each configuration was limited to three replicates (*n* = 3). While this is a typical sample size for exploratory studies on composite mechanics due to material and manufacturing constraints, it imposes limitations on the statistical robustness of the data compared to large-population studies. However, the reliability of the observed trends is supported by the high consistency of the experimental data. As listed in Table 5, the Coefficient of Variation (CV) for the ultimate compressive strength is consistently low, ranging from 2.0% to 4.5% across all configurations. This low scatter suggests that the manufacturing quality and testing procedures were stable. Furthermore, the trends discussed below—specifically the spacing threshold effect—are systematic across multiple orientation groups and are corroborated by the finite element predictions. Therefore, despite the limited sample size, the reported failure mechanisms and comparative trends are considered statistically representative of the material behavior.

**Figure 12:**
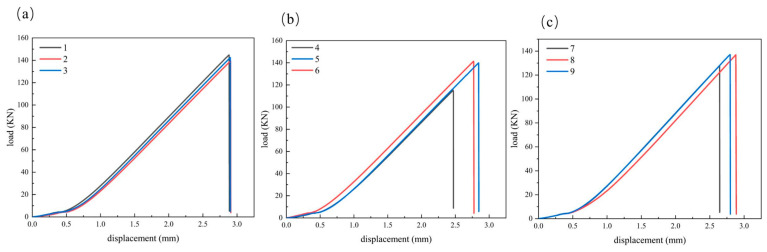
(**a**) Load–displacement diagram of longitudinal opening specimen. (**b**) Load–displacement diagram of transverse opening specimen. (**c**) Load–displacement diagram of 45° opening specimen.

The compression test load–displacement curves are presented in Figure 12.

The side clamping of the anti-buckling fixture may introduce additional transverse compression, which can slightly increase the measured ultimate strength compared with specimens tested with unsupported edges. Nevertheless, since all configurations were tested under identical boundary conditions, the relative trends reported herein remain valid.

Using experimental compression data, we systematically evaluated how inter-hole spacing and two-hole orientation influence the compressive properties of non-crimp fabric (NCF) composite laminates.

Holding the two-hole orientation constant, we compared the effect of inter-hole spacing. The results indicate that two-hole orientation and inter-hole spacing primarily influence failure initiation and ultimate load capacity, with a minimal effect on the linear-elastic regime. The elastic modulus of the three groups of specimens is in the range of 48.55–48.79 GPa, and the elastic modulus difference of the specimens with different hole spacing and two-hole orientation is not more than 1%. It is important to clarify that the elastic modulus values reported in Table 5 represent the apparent compressive stiffness of the laminate under the specific boundary conditions of the anti-buckling fixture. Since the fixture suppresses transverse Poisson expansion (ε_yy_ ≈ 0), the measured modulus is theoretically higher than the intrinsic uniaxial modulus (E_xx_) obtained from standard unconstrained coupons. The relationship is governed by E_apparent_ ≈ E_xx_/(1 − ν_xy_ν_yx_). Despite this boundary effect, the apparent stiffness remained remarkably consistent across all nine configurations, confirming that the presence of double-hole defects has a negligible impact on the global elastic response compared to the boundary constraints. Under the same opening direction, the average compressive strength Rm of the longitudinal opening is 290.5 MPa, 278.5 MPa, and 282.5 MPa when the hole spacing is 12 mm, 18 mm, and 30 mm, respectively. With two-hole orientation held constant (longitudinal), increasing inter-hole spacing from 12 to 18 mm results in a 4.1% reduction in the average OHC strength Rm, whereas a further increase from 18 to 30 mm leads to a 1.4% increase. The Rm corresponding to the transverse opening specimens is 228.5 MPa, 280 MPa, and 279.5 MPa when the hole spacing is 12 mm, 18 mm, and 30 mm, respectively. When the hole spacing increases from 12 mm to 18 mm, the compressive strength increases by 22.5%. When the hole spacing increases from 18 mm to 30 mm, the compressive strength is basically unchanged. The Rm corresponding to the 45° two-hole orientation opening specimen is 253.5 MPa, 273.5 MPa, and 271.5 MPa when the hole spacing is 12 mm, 18 mm, and 30 mm, respectively. The compressive strength increases by 7.9% when the hole spacing increases from 12 mm to 18 mm, and the compressive strength decreases by 0.7% when the hole spacing increases from 18 mm to 30 mm. In general, the increase in the hole spacing from 12 mm to 18 mm in the transverse and 45-degree direction can significantly weaken the inter-hole coupling and improve the strength, while the compressive strength of the longitudinal opening specimen decreases slightly. When the hole spacing of the specimens in the three opening directions continues to increase to 30 mm, the strength change usually does not exceed 2%, indicating that the strength is not sensitive to the influence of the hole spacing when s/D ≥ 3.

At fixed interpole spacing, we compared specimens with longitudinal, transverse, and 45° two-hole orientations. The results are as follows: When the hole spacing is 12 mm (s/D = 2), the strength order is as follows: the longitudinal opening specimen (290.5 MPa) is higher than the 45° opening specimen (253.5 MPa), and both are higher than the transverse opening specimen (228.5 MPa). Compared with the longitudinal opening specimen, the OHC strength of the transverse opening specimen and the 45° direction opening specimen is reduced by 21.3% and 12.7%, respectively, and the compressive strength of the 45° direction opening specimen is 9.9% higher than that of the transverse opening specimen, indicating that the direction effect is the strongest and the transverse opening is the most unfavorable. When the hole spacing is 18 mm (s/D = 3), the OHC strength of the transverse opening specimen (280.0 MPa) and the longitudinal opening specimen (278.5 MPa) is similar, which is higher than that of the 45° direction opening specimen (273.5 MPa). The difference between the compressive strength of the transverse opening specimen and the longitudinal opening specimen is negligible (0.54%). The 45° direction opening specimen is 1.8% lower than the longitudinal opening specimen, and the strength difference affected by the opening direction is significantly reduced. When the hole spacing is small (s/D = 2), the sensitivity of the opening direction is strong, and the transverse opening is the most unfavorable, while the difference in the opening directions is small when the hole spacing is large (s/D = 3). When the hole spacing is 30 mm (s/D = 5), the compressive strength of the longitudinal opening specimen (282.5 MPa) is close to the compressive strength of the transverse opening specimen (279.5 MPa), which is higher than the compressive strength of the 45° direction opening specimen (271.5 MPa). The difference between the compressive strength of the transverse opening specimen and the compressive strength of the longitudinal opening specimen is 1.1%. The compressive strength of the 45° direction opening specimen is 3.9% lower than that of the longitudinal opening specimen. The direction effect is further weakened, and the difference converges to several percentage points. The elastic modulus is likewise insensitive to the opening direction at fixed spacing, with differences not exceeding 1%.

The above trend is consistent with the damage mechanism: when the hole spacing is small (12 mm), the coupling phenomenon of the stress concentration area of the two holes is obvious, and the inter-hole ligament bears the superimposed transverse compressive stress and shear stress, which makes it easy to form the cross-hole main shear band and the penetration delamination, so the strength is low. When the hole spacing increases to 18 mm and above, the coupling phenomenon is significantly weakened, the damage tends to be localized at the trailing edge of the respective holes, and the load-carrying capacity is restored to the level of a single hole, so the performances of 18 mm and 30 mm are close. For longitudinal openings, although part of the 0° primary fiber path is interrupted, the central region still benefits from the synergy of continuous 0° fibers and off-axis plies (e.g., ±45°); hence, the ultimate load-bearing capacity and strength are generally higher than in the other orientations. A transverse opening directly severs the main load-bearing fiber bundles. The short-span ligament is more susceptible to shear failure and out-of-plane buckling, yielding strengths slightly lower than the 45° orientation. Although a 45° opening weakens portions of the ±45°/90° plies, it does not sever the primary 0° load path; the oblique crack path is also longer, so its strength typically exceeds that of the transverse orientation. It is worth noting that in the longitudinal opening, the increase in the hole spacing is equivalent to the elongation of the axial ligament in the load direction and the weakening of the constraint. The micro-buckling of the near-hole fiber and the delamination between the layers are easier to start, which is manifested as the strength of 12 mm being slightly higher than 18 mm and 30 mm; that is, a slight decrease occurs in the plateau region. In summary, increasing the spacing from s/D = 2 to 3 effectively weakens hole-to-hole coupling, bringing the compressive strength into a plateau region, while the elastic modulus remains essentially constant over the examined range.

### 5.3. Finite Element Analysis

Experimental observations under compressive loading revealed the specimens’ failure modes, and finite element simulations further corroborated the influence of the preformed openings on the material response.

**Figure 13:**
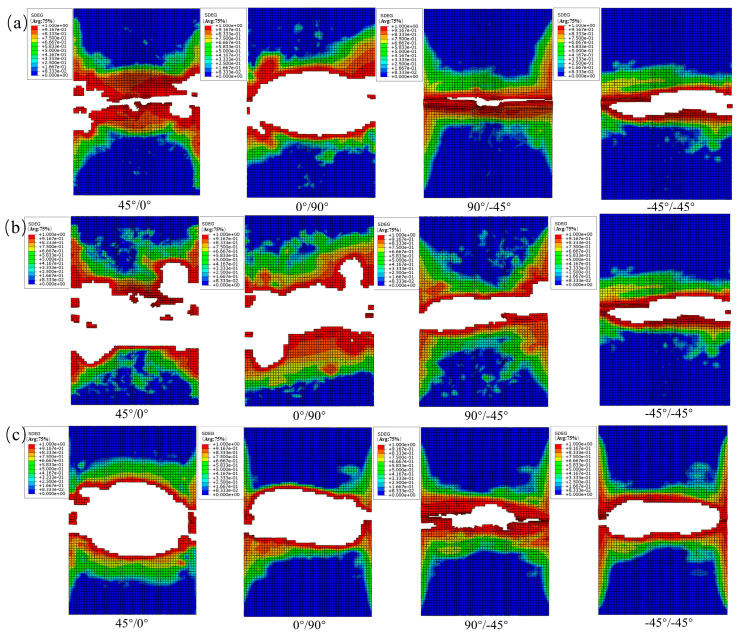
(**a**) SDEG damage contours for the transverse opening orientation (s = 12 mm); (**b**) SDEG damage contours for the transverse opening orientation (s = 18 mm); (**c**) SDEG damage contours for the transverse opening orientation (s = 30 mm).

From Figure 13, the interlaminar damage variable (SDEG) obtained from the cohesive elements for the transversely perforated specimens shows the following. In the contour maps of each interface, red (high-damage) regions first appear in the load-bearing ligament adjacent to the hole edge and develop into two nearly symmetric bands aligned with the loading direction, indicating mixed-mode-dominated delamination. As the inter-hole spacing increases, delamination between the holes transitions from ligament spanning to edge localized; both the peak SDEG and the length of equal-damage (isodamage) contours within the inter-hole ligament decrease markedly. The 45°/0° and 90°/−45° interfaces are the more damage-sensitive interfaces: the peak SDEG in the inter-hole ligament decreases substantially with increasing spacing. By contrast, at the 0°/90° and −45°/−45° interfaces, damage becomes more strongly localized at the hole edge.

**Figure 14:**
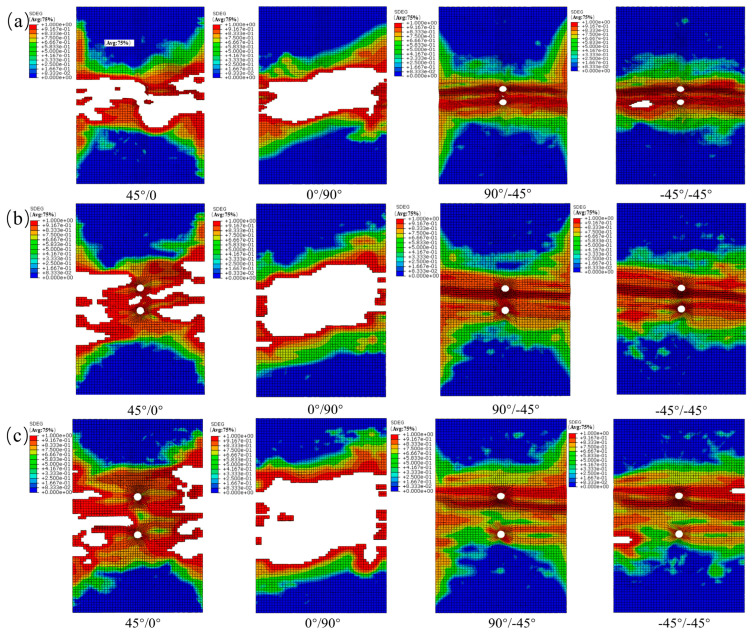
(**a**) SDEG damage contours for the longitudinal (load-aligned) opening configuration (s = 12 mm); (**b**) SDEG damage contours for the longitudinal (load-aligned) opening configuration (s = 18 mm); (**c**) SDEG damage contours for the longitudinal (load-aligned) opening configuration (s = 30 mm).

From Figure 14, the interlaminar damage variable (SDEG) obtained from the cohesive elements for the longitudinally perforated specimens shows the following. With increasing inter-hole spacing, the evolution of failure modes is similar to the transverse case: strong interaction and ligament-spanning delamination at small spacing transition to edge-localized damage as spacing increases. The 45°/0° and 0°/90° interfaces are the more damage-sensitive planes: they initiate the earliest continuous, ligament-spanning delamination and maintain elongated high-SDEG bands aligned with the loading direction (horizontal in the figures). By contrast, the −45°/−45° interface exhibits greater resistance to delamination (lower SDEG and more localized damage).

**Figure 15:**
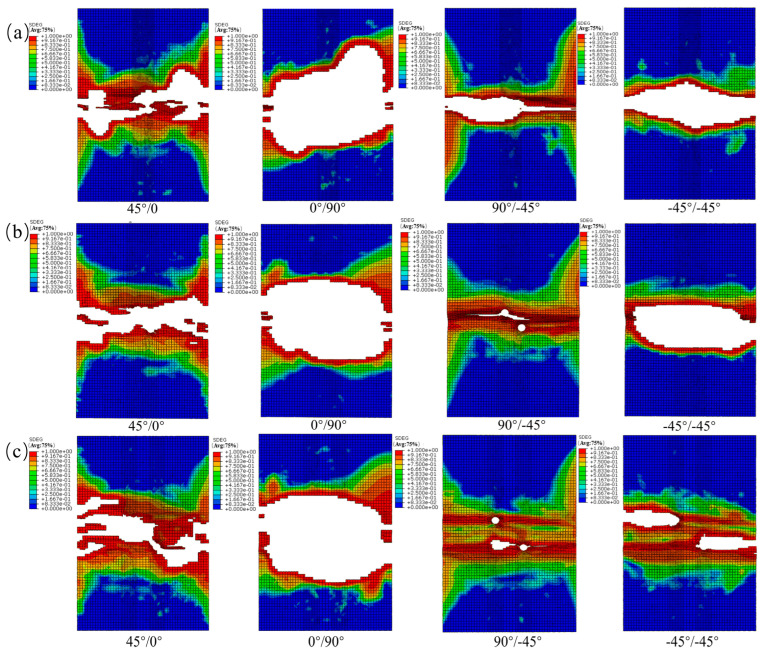
(**a**) SDEG damage contours for the 45° (off-axis) opening orientation (s = 12 mm); (**b**) SDEG damage contours for the 45° (off-axis) opening orientation (s = 18 mm); (**c**) SDEG damage contours for the 45° (off-axis) opening orientation (s = 30 mm).

From Figure 15, damage initiates at the facing (inner) edges of the two holes and develops into an oblique bridging band along the line connecting the holes. With increasing load, this band widens and coalesces to penetrate the ligament. Accompanied by pinnate branching and terminal bifurcation, the inter-hole ligament evolves toward highly damaged, near-saturated states at higher loads. The damage distributions predicted by the finite element model agree well with the experimentally observed failure patterns.

**Figure 16:**
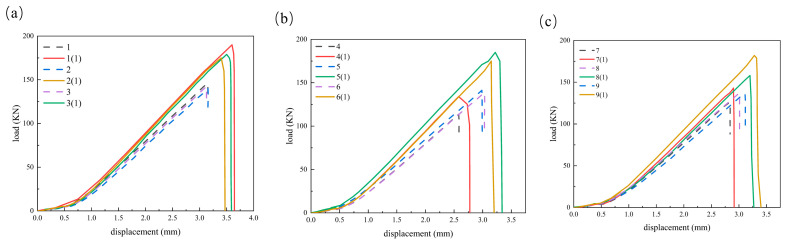
(**a**) Load–displacement comparison diagram of longitudinal opening specimen. (**b**) Load–displacement comparison diagram of 45° opening specimen. (**c**) Load–displacement comparison diagram of 45° opening specimen.

Building on the experimental trends summarized in Section 5.2 (Figure 12)—namely that inter-hole spacing and orientation chiefly affect failure initiation and ultimate strength, whereas the linear-elastic stiffness remains essentially unchanged—we now examine the simulation–test correspondence. As shown in Figure 16, the FEA curves exhibit excellent agreement with the experimental data, accurately reproducing the characteristic linear-brittle response observed in NCF laminates. Both numerical and experimental results show a linear stress–strain relationship up to the ultimate load, followed by a sharp post-peak degradation. This confirms that the model correctly captures the sudden nature of the failure, which is driven by the rapid propagation of fiber kinking and shear damage once the critical load is reached, rather than a gradual softening process. Remaining numerical–experimental differences are within 16% over the examined range. This level of agreement is consistent with the progressive-damage framework employed (3D Hashin + modified matrix criterion with shear coupling coupled with a cohesive-zone model for delamination), which reproduces the observed damage evolution under compression.

## 6. Conclusions

Opening orientation and inter-hole spacing have a significant effect on the OHC strength of NCF laminates, while the apparent elastic stiffness remains essentially insensitive to these geometric parameters. Experimental results indicate that hole spacing exhibits a threshold-like effect on compressive strength. For transverse and 45° orientations, increasing the spacing ratio (s/D) from 2 to 3 yields a pronounced strength gain, whereas further increases beyond this threshold provide diminishing returns. In contrast, the longitudinal configuration shows a slight strength decrease as spacing increases, with failure modes becoming increasingly localized. Specifically, at small spacing (s = 12 mm), directional effects are strongest, with the transverse configuration being the weakest due to ligament instability. Furthermore, the stitching defects in NCF materials were found to promote damage coalescence between holes. The finite element framework, coupling the 3D Hashin criterion and a modified matrix criterion incorporating shear coupling with a cohesive-zone model, successfully reproduced the load–displacement response and failure limits. The model accurately captured the nonlinear damage evolution mechanisms preceding structural collapse, as well as the transition from ligament-spanning delamination to localized failure, validating its effectiveness in predicting the failure mechanisms of multi-hole NCF laminates.

## Figures and Tables

**Table 1:** Material mechanical properties.

Fiber Type	Tensile Strength (MPa)	E (GPa)	Elongation at Break (%)	Density (g·cm^−3^)
CCF300 3k	3900	220	1.86	1.78

**Table 2:** Typical specimen numbers.

Sample ID	Center-to-Center	Opening Direction
1	12 mm	long direction
2	18 mm	long direction
3	30 mm	long direction
4	12 mm	cross direction
5	18 mm	cross direction
6	30 mm	cross direction
7	12 mm	45° direction
8	18 mm	45° direction
9	30 mm	45° direction

**Table 3:** Damage evolution parameters.

Parameter	Parameter Value
Longitudinal tensile strength X_t_	3900 MPa
Longitudinal compressive strength X_C_	2340 MPa
Transverse tensile strength Y_t_	70 MPa
Transverse compressive strength Y_C_	140 MPa
Through-thickness tensile strength Z_t_	70 MPa
Through-thickness compressive strength Z_C_	140 MPa
Shearing strength S_12_	70 MPa
Shearing strength S_13_	70 MPa
Shearing strength S_23_	56 MPa
Longitudinal tensile fracture energy G_1t_	40 kJ/m^2^
Longitudinal compression fracture energy G_1c_	100 kJ/m^2^
Transverse tensile fracture energy G_2t_	0.5 kJ/m^2^
Transverse tensile fracture energy G_2c_	1.5 kJ/m^2^

**Table 4:** Failure modes at different locations.

Position	Main Failure Models
Hole edge area	Bearing failure and shear failure
Inter-hole area	Shear failure and interlaminar delamination
Plate edge	Interlayer delamination and fiber buckling/breaking
Plate side	Interlayer delamination and matrix cracking

**Table 5:** Summary of experimental data.

Number	Fm (kN)	Rm (MPa)	E (GPa)	c–c Spacing	Hole Alignment	CV of Rm (%)
1	145.47 ± 4.5	291 ± 9.0	48.857 ± 0.73	12 mm	Longitudinal	3.1%
2	139.45 ± 5.9	279 ± 11.7	48.559 ± 0.68	18 mm	Longitudinal	4.2%
3	141.20 ± 3.9	282 ± 7.9	48.826 ± 0.58	30 mm	Longitudinal	2.8%
4	114.42 ± 5.2	229 ± 10.3	48.592 ± 0.97	12 mm	Transverse	4.5%
5	139.91 ± 2.8	280 ± 5.6	48.665 ± 0.49	18 mm	Transverse	2.0%
6	139.56 ± 4.2	280 ± 8.4	48.798 ± 0.78	30 mm	Transverse	3.0%
7	126.69 ± 4.8	253 ± 9.6	48.865 ± 0.83	12 mm	45° orientation	3.8%
8	136.53 ± 3.5	273 ± 7.1	48.657 ± 0.53	18 mm	45° orientation	2.6%
9	137.13 ± 4.1	274 ± 8.2	48.611 ± 0.63	30 mm	45° orientation	3.0%

## Data Availability

The data presented in this study are openly available in Analysis of Residual Compressive Strength in 3D Four-Directional Braided Composites After Hygrothermal Aging at https://doi.org/10.3390/ma18061368 (accessed on 18 January 2026).

## Supplementary Materials

The following supporting information can be downloaded at: https://www.mdpi.com/article/10.3390/ma19030495/s1, File S1: Damage Criterion.
