# Supplementary material for: Compressive Failure Mechanisms of NCF Laminates with Double-Hole Defects
Source: Materials (Basel). 2026 Jan 26;19(3):495. doi: 10.3390/ma19030495 (PMC12898083; doi:10.3390/ma19030495)
Supplement: Supplementary file 1 [file materials-19-00495-s001.zip › materials-4096454-supplementary.pdf]

*4.3. Damage Criterion*

The failure criteria for fiber tension and compression and matrix crushing follow the 3D Hashin criterion; matrix-dominated failure under multiaxial shear–compression is treated using a quadratic strain-based criterion with shear-compression coupling, extending the classical Hashin approach in the VUMAT.

Hashin governs fiber-dominated modes. Under $e_{22}+e_{33}<0$ (matrix compression state), a modified quadratic expression incorporating a compression–shear coupling coefficient $\alpha$ is used to capture the strengthening effect of transverse compression on shear strength; when normal opening dominates ($e_{22}+e_{33}>0$), matrix failure degenerates to Hashin's matrix-tension form.

The stress-based Hashin criterion is as follows: fiber tensile failure (when the fiber direction strain > 0),

$$\left(\frac{\sigma_{11}}{X_T}\right)^2+\left(\frac{\sigma_{12}}{S_{12}}\right)^2+\left(\frac{\sigma_{13}}{S_{13}}\right)^2\geq 1 \tag{S2}$$

where $X_t$ is the tensile strength of the fiber, $S_{12}$, $S_{13}$ is the shear strength, $X_c$ is the compressive strength of the fiber.

Fiber compression failure (when fiber direction strain e11< 0)

$$\left(\frac{\sigma_{11}}{X_C}\right)^2\geq 1 \tag{S3}$$

Matrix compression failure criterion (when e22 + e33 < 0)

$$\left(\frac{\sigma_{22}+\sigma_{33}}{Y_C}\right)^2+\frac{\sigma^2{}_{23}-\sigma_{22}\sigma_{33}}{S^2{}_{23}}+\left(\frac{\sigma_{12}}{S_{12}}\right)^2+\left(\frac{\sigma_{13}}{S_{13}}\right)^2\geq 1 \tag{S4}$$

Matrix tensile failure criterion (when $e_{22}+e_{33}>0$)

$$\left(\frac{e_{22}}{e_{2t}}\right)^2+\alpha\left(\frac{e_{23}}{e^*{}_{23}}\right)^2\geq 1 \tag{S5}$$

In the formula: $e_{22}$ is the transverse principal strain, $e_{2t}$ is the tensile strain limit of the matrix, e23 is the transverse shear strain, and $\gamma_{23}$ is the shear limit strain. Notably, $\alpha$ represents the shear-compression coupling coefficient, physically reflecting the internal friction angle of the matrix. This modification incorporates the strengthening effect of transverse compression on shear strength (analogous to the Mohr-Coulomb internal friction effect). Unlike standard Hashin criteria, which often neglect the suppression of shear band formation by normal compressive stress, the VUMAT implements this coupling term. This is critical for NCF laminates where resin-rich pockets at stitching points make the matrix response highly sensitive to the multiaxial stress state.

To accurately capture delamination in laminates under compression, the intralaminar progressive-damage model was augmented by introducing an interlaminar cohesive-zone formulation based on a traction–separation law, and, enforcing energy consistency, the corresponding damage-evolution law was derived from the mode-dependent fracture energies.

### *4.4. Traction-Separation Relationship and Definition of Damage Variable*

At the interface of the laminated material, it is assumed that there are normal and tangential separation displacements, defined by:

Normal separation displacement:

$$\delta_n = \langle \upsilon_n \rangle \tag{S6}$$

Tangential separation displacement:

$$\delta_s = \sqrt{(\upsilon_{s1}{}^2 + \upsilon_{s2}{}^2)} \tag{S7}$$

Effective separation displacement:

$$\delta_{ext} = \sqrt{(\delta_n{}^2 + \beta^2 * \delta_s{}^2)} \tag{S8}$$

where ⟨·⟩ denotes the Macaulay bracket, denotes a contribution only when $\delta_n > 0$ is opened; $\beta$ is the shear weight coefficient and is typically set to 1.

The interface traction-separation relationship satisfies the following expression:

$$t = (1 - d_{\text{int}}) K_0 \delta \tag{S9}$$

$$K_0 = diag(K_n, K_s, K_s) \tag{S10}$$

where: t is the traction vector ($t_n$, $t_{s1}$, $t_{s2}$); $K_0$ is the initial interface stiffness matrix; $d_{\text{int}} \in [0,1]$ for inter-layer variable damage, 0 means intact and 1 means complete failure.

#### 4.4.1. Damage Initiation Criterion

The nominal stress quadratic criterion is used for damage initiation:

$$\left\langle \frac{t_n}{\sigma_{\max}} \right\rangle^2 + \left( \frac{t_s}{\tau_{\max}} \right)^2 + \left( \frac{t_t}{\tau_{\max}} \right)^2 = 1 \tag{S11}$$

The two tangential stresses are combined into a "shear":

$$t_{shear} = \sqrt{(t_s{}^2 + t_t{}^2)} \tag{S12}$$

Damage initiation criterion:

| | |
|---|---|
| $$\left\langle \frac{t_n}{\sigma_{\max}} \right\rangle^2 + \left( \frac{t_{shear}}{\tau_{\max}} \right)^2 = 1$$ | (S13) |

Defining the effective traction $t_n^0 = \sigma_{\max}$, $t_s^0 = \tau_{\max}$, the failure index is:

| | |
|---|---|
| $$\phi = \left( \frac{\langle t_n \rangle}{t_n^0} \right)^2 + \left( \frac{t_{\text{shear}}}{t_s^0} \right)^2 \geq 1$$ | (S14) |

When $\phi$ ≥1, the damage begins to evolve.

Among them, $t_n^0$ and $t_s^0$ are the normal and shear peak strength of the interface, respectively.

Before the damage starts, it is linear elasticity:

| | |
|---|---|
| $$t_n = K_n \langle \delta_n \rangle$$ | (S15) |

| | |
|---|---|
| $$t_{shear} = K_s \delta_s$$ | (S16) |

| | |
|---|---|
| $$\delta_n^0 = \frac{\sigma_{\max}}{K_n} = \frac{t_n^0}{K_n}, \delta_s^0 = \frac{\tau_{\max}}{K_s} = \frac{t_s^0}{K_s}$$ | (S17) |

Damage activation condition:

| | |
|---|---|
| $$\left( \frac{K_n \langle \delta_n \rangle}{t_n^0} \right)^2 + \left( \frac{K_s \delta_s}{t_s^0} \right) = 1 \Leftrightarrow \left( \frac{\langle \delta_n \rangle}{\delta_n^0} \right)^2 + \left( \frac{\delta_s}{\delta_s^0} \right) = 1$$ | (S18) |

| | |
|---|---|
| $$\delta_0 = \sqrt{(\delta_n^0)^2 + \beta^2 (\delta_s^0)^2} = \sqrt{(\frac{t_n^0}{K_n})^2 + \beta^2 (\frac{t_s^0}{K_s})^2}$$ | (S19) |

Once damage initiates, the initial equivalent displacement $\delta_0$ is recorded.

4.4.2. Damage Evolution and Energy Consistency

Interface separation vector:

| | |
|---|---|
| $$\delta = [\delta_n, \delta_s, \delta_t]^T$$ | (S20) |

Traction vector:

| | |
|---|---|
| $$t = [t_n, t_s, t_t]^T$$ | (S21) |

Define equivalent separation and equivalent traction:

| | |
|---|---|
| $$\delta_{eff} = \sqrt{(\frac{\langle \delta_n \rangle}{\delta_n^0})^2 + \beta_s^2(\frac{\delta_s}{\delta_s^0})^2 + \beta_t^2(\frac{\delta_t}{\delta_t^0})^2}$$ | (S22) |

$$t_{eff} = \sqrt{(\frac{t_n}{t_n^0})^2 + \beta_s^2(\frac{t_s}{t_s^0})^2 + \beta_t^2(\frac{t_t}{t_t^0})^2} t^*$$

(S23)

t* is the benchmark of dimensionless stress. When $\phi$ =1, $\delta_0 = \delta_{eff}$, $t_0 = t_{eff}$.

Linear softening envelope (from $(\delta_0, t_0)$ linear back to $(\delta_f, 0)$):

| | |
|---|---|
| $$t_{eff}(\delta) = t_0(1 - \frac{\delta - \delta_0}{\delta_f - \delta_0})$$, | (S24) |

where $\delta_0 < \delta < \delta_f$.

Energy consistency: fracture energy is equal to the area of softening section.

| | |
|---|---|
| $$G_c = \int_{\delta_0}^{\delta_f} t_{eff}(\delta) d\delta = \frac{1}{2} t_0(\delta_f - \delta_0)$$ | (S25) |

Energy-based damage evolution:

| | |
|---|---|
| $$\delta_f = \delta_0 + \frac{2G_c}{t_0}$$ | (S26) |

When the equivalent traction of the softening section degenerates into $t_{eff} = (1-d)t_0$, damage evolution can be obtained:

| | |
|---|---|
| $$d(\delta_{eff}) = \begin{cases} 0, \delta_{eff} \le \delta_0 \\ \frac{\delta_{eff} - \delta_0}{\delta_f - \delta_0}, \delta_0 < \delta_{eff} < \delta_f \\ 1, \delta_{eff} > \delta_f \end{cases}$$ | (S27) |

4.4.3. Mixed-Mode Failure Energy Criterion

Using the Benzeggagh–Kenane (BK) criterion, the dimensionless interpolation of the mixed-mode fracture energy is performed by a single-parameter power function, and the following is obtained:

| | |
|---|---|
| $G_c^{mix} = G_{\mathrm{Ic}} + (G_{\mathrm{IIc}} + G_{\mathrm{IIIc}} - G_{\mathrm{Ic}})(\frac{G_{\mathrm{II}} + G_{\mathrm{III}}}{G_{\mathrm{I}} + G_{\mathrm{II}} + G_{\mathrm{III}}})^{\eta}$ | (S28) |

In the formula: $G_c^{mix}$ is the critical fracture energy; $G_{\mathrm{I}}, G_{\mathrm{II}}, G_{\mathrm{III}}$ the energy release rate; $G_{\mathrm{Ic}}, G_{\mathrm{IIc}}, G_{\mathrm{III}c}$ is the modal critical value.
